# *HvGBSSI* mutation at the splicing receptor site affected RNA splicing and decreased amylose content in barley

**DOI:** 10.3389/fpls.2022.1003333

**Published:** 2022-09-23

**Authors:** Xiuqin Feng, Md. Mostafijur Rahman, Qian Hu, Bang Wang, Hassan Karim, Carlos Guzmán, Wendy Harwood, Qiang Xu, Yazhou Zhang, Huaping Tang, Yunfeng Jiang, Pengfei Qi, Mei Deng, Jian Ma, Jingyu Lan, Jirui Wang, Guoyue Chen, Xiujin Lan, Yuming Wei, Youliang Zheng, Qiantao Jiang

**Affiliations:** ^1^State Key Laboratory of Crop Gene Exploration and Utilization in Southwest China, Sichuan Agricultural University, Chengdu, Sichuan China; ^2^Triticeae Research Institute, Sichuan Agricultural University, Chengdu, Sichuan, China; ^3^Departamento de Genética, Escuela Técnica Superior de Ingeniería Agronómica y de Montes, Universidad de Córdoba, Cordoba, Spain; ^4^John Innes Center, Norwich Research Park, Norwich, United Kingdom

**Keywords:** *Hordeum vulgare*, EMS, *Wx-1*, abnormal RNA splicing, starch-binding capacity, starch properties

## Abstract

Granule-bound starch synthase I (HvGBSSI) is encoded by the barley *waxy* (*Wx-1*) gene and is the sole enzyme in the synthesis of amylose. Here, a *Wx-1* mutant was identified from an ethyl methane sulfonate (EMS)-mutagenized barley population. There were two single-base mutations G1086A and A2424G in *Wx-1* in the mutant (M2-1105). The G1086A mutation is located at the 3′ splicing receptor (AG) site of the fourth intron, resulting in an abnormal RNA splicing. The A2424G mutation was a synonymous mutation in the ninth intron. The pre-mRNA of *Wx-1* was incorrectly spliced and transcribed into two abnormal transcripts. The type I transcript had a 6 bp deletion in the 5′ of fifth exon, leading to a translated HvGBSSI protein lacking two amino acids with a decreased starch-binding capacity. In the type II transcript, the fourth intron was incorrectly cleaved and retained, resulting in the premature termination of the barley *Wx-1* gene. The mutations in the *Wx-1* decreased the enzymatic activity of the HvGBSSI enzyme and resulted in a decreased level in amylose content. This work sheds light on a new *Wx-1* gene inaction mechanism.

## Introduction

Barley (*Hordeum vulgare* L.) is an excellent source of complex carbohydrates and is globally the fourth most cultivated and consumed grain after corn, rice, and wheat (Newton et al., 2011). The barley grain is an important source of starch, and accounts for 60%–75% of the grain weight (Asare et al., 2011). Starch can be divided into amylose and amylopectin according to their molecular structures (Maningat et al., 2009). Amylose contains hundreds of glucose units and is a linear molecule connected by α-(1,4)-linkage bonds, whereas amylopectin contains thousands of glucose units and forms a polymer with a high molecular weight by connecting α-(1,4)-linkage bonds and α-(1,6)-linkage bonds. The content and ratio of amylose and amylopectin considerably affect the physicochemical properties of starch and therefore determine the edible, feeding, and processing quality of barley (Li et al., 2014). The amylose content in waxy barley grain is lower than 10% whereas that in non-waxy barley grain is 20%–30% (Domon et al., 2002; Sullivan et al., 2013). Moreover, waxy barley starch has the advantages such as high viscosity, good cooking resistance, and ductility owing to its low amylose content. Thus, it is used as an ingredient in several foods, such as bread, biscuits, desserts, and special baby foods, as well as in thickeners, expansion agents, and adhesives (Brennan and Cleary, 2005).

Granule-bound starch synthase I (GBSSI) is the sole enzyme in the synthesis of amylose and is closely associated with amylose contents (Rahman et al., 2000). As a granule-bound starch synthase, GBSSI is able to catalyze the synthesis of amylose only when it binds to starch granules (Mason Gamer et al., 1998; Valdez et al., 2008; Liu et al., 2015). HvGBSSI is encoded by the *waxy* (*Wx-1*) gene that is located on chromosome 7H in barley, and HvGBSSI protein contains 603 amino acids with molecular weight of approximately 60 kDa (Rohde et al., 1988; Domon et al., 2002). The deletion or mutation of *Wx-1* in rice, maize, wheat, and barley leads to a decrease in GBSSI activity, thereby decrease in a amylose content (Olsen and Purugganan, 2002). A previous study reported that the deletion of a fragment containing the TATA box and the transcription initiation site in the *Wx-1* promoter region has significantly decreased the *HvGBSSI* expression and amylose content in barley (Domon et al., 2002). Mutations in important amino acids of barley HvGBSSI may block the synthesis of amylose. For instance, a previously published study showed that an amino acid substitution in HvGBSSI resulted in waxy barley (Patron et al., 2002).

For the proper splicing of pre-mRNAs, the pre-mRNA spliceosome recognizes three elements, the 5′ splicing site, the 3′ splicing site, and the branching site of the pre-mRNA sequence (Reddy, 2010; Duque, 2014). The U1 small nuclear ribonucleoproteins (snRNP) complex formed by U1 snRNP and protein binds to the 5′ splicing site with a generally conserved GU sequence, and subsequently, the U2 snRNP auxiliary factor (U2AF) binds to the 3′ splicing site with a generally conserved AG sequence, thereby promoting the binding of U2 snRNP to the branching sequence. Next, U4 snRNP, U5 snRNP, and U6 snRNP are recruited to remove introns and exon linkage (Schmidt and Kirschner, 2013). Exonic splicing enhancers (ESEs) have been identified as short oligonucleotide sequences that often enhance exon recognition in splicing through the action of proteins of the serine/arginine (SR)-rich protein family (Graveley, 2000; Cartegni et al., 2002).

Ethyl methane sulfonate (EMS) is a commonly used chemical that induces mutations and has been widely used in crops, such as maize, rice, and wheat (Chen et al., 2012; Serrat et al., 2014; Lu et al., 2017). The mutant lines caused by EMS can be used directly for breeding without any gene-modification controversies (Henikoff and Comai, 2004). In this study, we created an EMS-mutagenized barley population, and identified a mutant, M2-1105, with a base mutation at fourth intron splicing receptor site, leading to a near-waxy phenotype with decreased levels of amylose. This work reports the characterization of a novel molecular mechanism of *Wx-1* inactivation and its effects on the starch properties of the barley mutant line.

## Materials and methods

### Materials

Barley cv. Golden Promise (GP) seeds were mutagenized using EMS as previously published methods (Slade et al., 2005). Briefly, about 1,500 dried seeds were divided into two groups, with each group wrapped in cotton yarn and soaked in a 100 ml EMS solution for about 18 h. To ensure that the germination rate of the mutated barley seeds was about 50%, germination experiments were performed in 0.3%–0.9% EMS concentrations, from which the most suitable EMS concentration was selected. After EMS treatment, the seeds were washed with tap water for 3 h. The EMS-treated seeds (M1) germinated in the greenhouse in November 2017, then were planted in the field in November 2017. The M2-generation seeds were harvested from one ear of each M1 plant in June 2018.

### Electrophoresis of total protein and GBSSI protein

Granule-bound starch synthase I protein and total protein extraction was performed as previously described (Yamamori and Endo, 1996; Hurkman and Tanaka, 2007; Hebelstrup et al., 2017). Total proteins and GBSSI protein were separated using sodium dodecyl sulfate-polyacrylamide gel electrophoresis (SDS-PAGE) in a discontinuous Tris–Hcl-SDS buffer system (pH: 6.8/8.8) with a polyacrylamide concentration of 12% (w/v; Ayala et al., 2015). Electrophoresis was performed at a constant current of 100 V/gel for 2.5 h immediately after the tracking dye had migrated off the gel. The total proteins and GBSSI proteins were detected *via* silver staining of the protein gels (Chevallet et al., 2007).

### Western blotting

The total protein extraction method was the same as described above. Protein and starch granules were extracted from the supernatant. Briefly, 60 mg of developmental endosperm was ground and mixed with a 200 μl of buffer (10 mM Tris–HCl, pH 8.0) solution and centrifuged at 8,000*g* for 5 min. Next, the supernatant was collected and centrifuged three times. The resulting pellet, containing starch granules, was then washed 10 times with the buffer solution. Protein isolation was performed using SDS-PAGE as described above, following which proteins were transferred to a polyvinylidene fluoride membrane. GBSSI proteins were detected using GBSSI antibodies from rabbit serum (ABclonal, Anhui, China) at a dilution of 1:1,000 as the primary antibody. Horseradish peroxidase-labeled goat anti-rabbit IgG(H + L) (11000; Beyotime, Shanghai, China) was used as the secondary antibody. The blots were visualized using BeyoECL Star (Beyotime, Shanghai, China; Hebelstrup et al., 2017).

### Cloning of *Wx-1* gene and transcript

Genomic DNA was extracted from the leaves of 2-week-old seedlings in M3-generation using the cetyltrimethylammonium bromide (CTAB) method (Murray and Thompson, 1980). To obtain *Wx-1* transcripts, seeds collected 15 days after flowering were selected for total RNA extraction using the Plant Genomic RNA Kit (BIOFIT, China). The PrimeScript™ reagent kit with gDNA Eraser (TAKARA, Dalian, China) was used for the reverse transcription of RNA. Based on the reference *Wx-1* gene (GenBank accession: AF486514) sequence in the NCBI database, the primers Wx-1-F and Wx-1-R were designed to amplify the DNA and cDNA of *Wx-1* (Supplementary Table S1). PCR amplification was performed using Phanta Max super-fidelity DNA Polymerase (Vazyme, Nanjing, China) to avoid sequence introduction errors. The PCR products were isolated on 1% agarose gel. All DNA fragments were recovered, purified, and ligated to pEasY-T1 (TransGen Biotech, Beijing, China). The DNA sequencing was conducted by a commercial company (Sangon Biotech, Shanghai, China). The final nucleotide sequence of each fragment was determined from the sequencing results of at least three independent clones.

### Sequence analysis

The nucleotide sequences were assembled and aligned using the DNAMAN software package (v. 9.0; Lynnon Biosoft). The gene sequences of *Wx-1* from wild barley GP and the mutant line M2-1105 were BLASTed and analyzed using ESEfinder3.0 webserver^1^ to identify local shear factors (Cartegni et al., 2003; Smith et al., 2006). Phyre2^2^ (Kelley et al., 2015) and the AlphaFold Protein Structure Database (Jumper et al., 2021) were used to predict the protein structure of HvGBSSI in the wild-type GP and mutant line M2-1105.

### Real-time quantitative PCR (qRT-PCR) analysis

The expression of *Wx-1* transcripts in M2-1105 and GP grains 15 days after flowering was determined by using qRT-PCR. Primers specific to *Wx-1* transcripts were designed (Supplementary Table S1), and the amplification was performed using ChamQ™ Universal SYBR^®^ qPCR Master Mix (Vazyme) on a CFX 96 Real-Time PCR System (Bio-Rad, Hercules, United States). The qRT-PCR data were analyzed using the CFX 96 Real-Time System, and the relative expression was calculated using the 2^−ΔΔC^_T_ method. Relative expression of the candidate genes was normalized using *β-Actin* and *GAPDH* as the reference genes.

### Prokaryotic expression and purification of proteins

The primers HvWx-1-PE-F and HvWx-1-PE-R were used to amplify the entire length of GP and Type I cDNA of mutant M2-1105 (Supplementary Table S1). The target gene fragment was amplified through PCR using the Phanta Max super-fidelity DNA Polymerase, and the expression vector pET-32 (a+) was digested with *Hin*d III and *Sac* I enzymes. The digested products were ligated with the T4 ligase, and the ligated mixtures were transformed into competent *Escherichia coli* DH5a cells. The recombinant constructs pET32(a+)-GP and pET32(a+)-Type I were transformed into *E. coli Rosetta* strain (DE3) competent cells. Single-cell colonies were inoculated in Luria-Bertani broth medium containing 100 μg ml^−1^ ampicillin at 37°C until an OD_600_ of approximately 0.4–0.6 was achieved. Next, 0.3 mM isopropyl-β-d-thiogalactoside (IPTG) was added to the culture, and the incubation was continued for 3–5 h. The culture was centrifuged at 4°C and 12,000*g* to obtain *E. coli* cells. The bacterial cells were then lysed with BugBuster (Novagen, San Diego, CA), and fusion proteins were purified on an Ni-NTA column according to the manufacturer′s instructions (Qiagen, Dusseldorf, Germany). The expressed proteins were detected by SDS-PAGE.

### Starch-binding assay

Starch-binding assay was performed as previously described (Liu et al., 2015). Purified recombinant GBSSI (final concentration of 5 μM) was added to a 10 μl 10% (w/v) corn starch suspension in 100 mM citrate–phosphate buffer (pH 5.0) up a final volume of 60 μl. Each mixture was incubated with gentle shaking (10 rpm) at 4°C for 1 h and centrifuged at 12,000*g* for 5 min at 4°C. The upper material was designated as “supernatant.” After treatment of the samples with the SDS buffer, the bound GBSSI was released from the starch granules using the SDS buffer, and these were designated as “bound” ones. The amount of GBSSI in each determined supernatant washed, and the bound complex was analyzed by Western blotting using an anti-GBSSI antibodies.

### Starch granule morphology

Starch granules were extracted as previously described (Zhang et al., 2022). A scanning electron microscope (SEM), ZEISS Gemini SEM300 (Carl ZEISS AG, Oberkochen, Germany), was used to analyze and record the morphology of the starch granules. The starch granules were evenly distributed on the tape stuck on the loading platform for observation. As per the instrument instruction, each sample was viewed under three different fields and the morphology of the starch granules was observed at 1,000× and 2,000× magnification.

### Starch granule size analysis

Starch granule size distribution was measured using the Mastersizer3000 laser diffraction particle size analyzer (Malvern Panalytical, England). Briefly, 140 mg of starch granules were suspended in 1 ml of anhydrous ethanol, and the starch granule suspension was mixed with 180 ml of distilled water in a beaker. After the shading degree reached the desired range of 12–17, the particle size distribution was measured using ultrasonic circulation for 2 min. The measurements were repeated three times for each sample. The volume and quantity of starch granules were determined using the theMastersizer 3000 operating software.

### Determination of GBSSI enzymatic activity, and total starch, amylose, resistant starch, and protein contents

Granule-bound starch synthase I enzymatic activity was determined using the GBSSI enzymatic activity assay kit (Solarbio Science & Technology, Beijing, China) according to the manufacturer′s instructions. Total starch, amylose, and resistant starch contents in GP and M2-1105 were determined using the total starch (AA/AMG) Assay Kit, Amylose/Amylopectin Assay Kit, and Resistant Starch Assay Kit (Megazyme, Wicklow, Ireland), respectively. Protein content was determined using the automatic Kjeldahl apparatus equipment (Foss Analytical Kjeltec™ 8400 series), as previously described (Yang et al., 2019). Each sample was analyzed in triplicate.

### Statistical analysis

Three replicates were used for each test. All data samples were expressed as means ± SD. One-way analysis of variance (ANOVA) was used to compare the differences between the values obtained from the mutant and wild-type lines. ANOVA was performed using IBM SPSS Statistics 26 (SPSS Inc., Chicago, IL, United States). *p* < 0.05 was considered statistically significant and *p* < 0.01 was considered extremely statistically significant.

## Results

### Identification of HvGBSSI protein mutant

**Figure 1:**
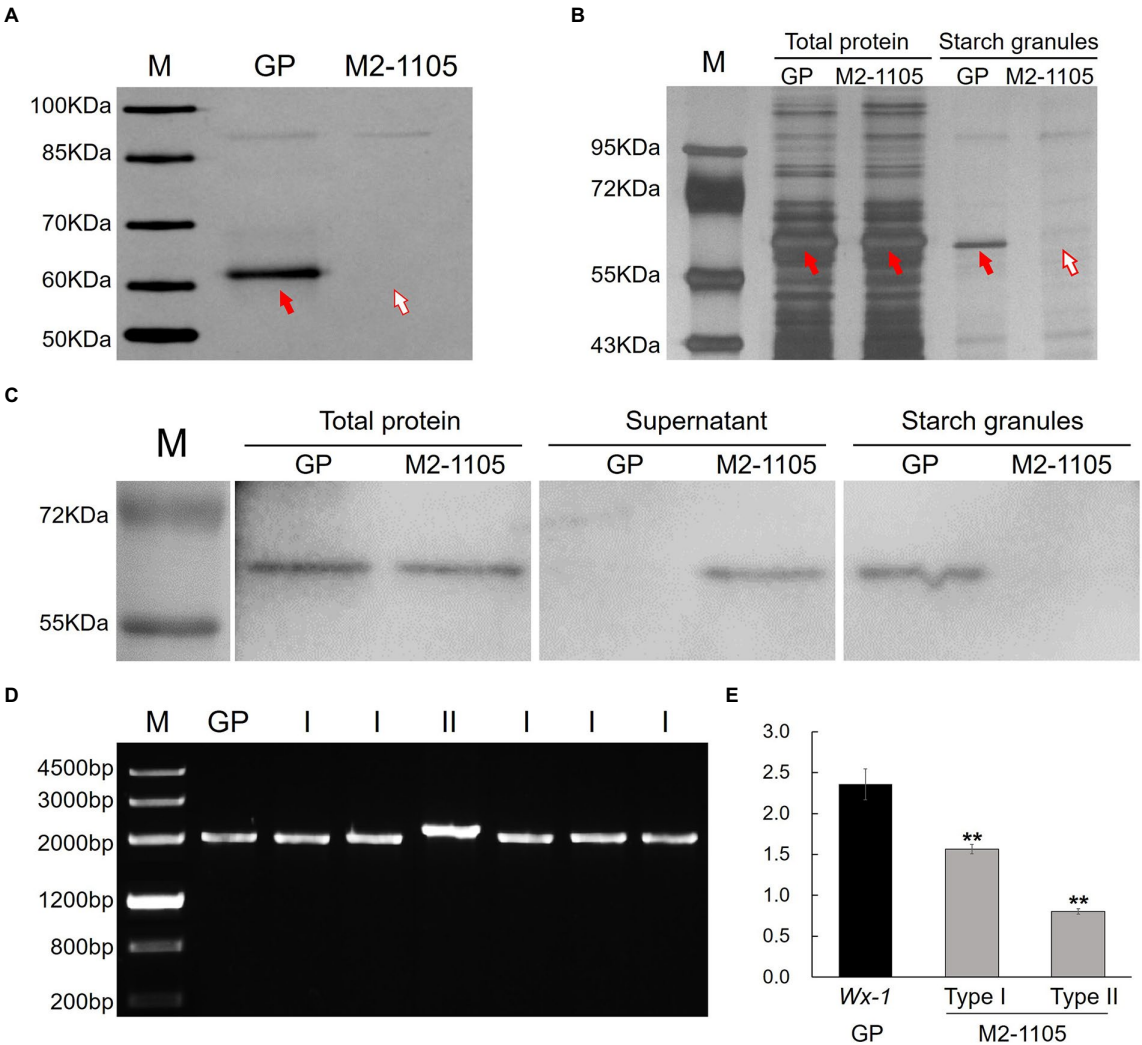
Identification of HvGBSSI protein and cDNA from the mutant M2-1105 line. **(A)** HvGBSSI protein was extracted from starch granules of mature grains of parental Golden Promise (GP) and the mutant M2-1105, and further analyzed by SDS-PAGE. The red arrow indicates the HvGBSSI protein. **(B)** Total protein and starch granules protein composition of GP and M2-1105 developing grains (15 days). **(C)** Western blotting to detect HvGBSSI protein as present in total, the supernatant, and starch granules of GP and M2-1105 developing seeds. Western blotting with HvGBSSI antiserum confirmed that HvGBSSI was strictly targeted to starch granules in the endosperm of GP, whereas it was mostly found in the supernatant of the endosperm cell fraction prepared from the M2-1105 mutant. **(D)** Amplification of transcripts of the *waxy* gene in mutant GP and the M2-1105 mutant. **(E)** The expression of the *Wx-1* gene and two types of transcripts relative to *β-Actin* and *GAPDH* 15 days after flowering. **Highly significant difference (*p* < 0.01).

Using SDS-PAGE, a mutant with defect in HvGBSSI expression in starch granules was identified in the M2 mutagenized population. The mutant, termed as M2-1105, lacked the corresponding band of HvGBSSI protein, in contrast with GP (Figure 1A). In terms of appearance, the seeds of M2-1105 were not different in length, width, fullness, or color from those of GP (Supplementary Figure S1).

We investigated the protein profiles in the developing grains of GP and the M2-1105 mutant. The HvGBSSI protein bands were present in the total proteins of M2-1105 as same with GP. In contrast, the HvGBSSI protein bands were not present in the protein profiles extracted from the M2-1105 starch granules but were present in those of GP (Figure 1B). Western blotting was then used to detect HvGBSSI protein in the total, the supernatant fraction, and purified starch granules from developing grains of GP and the M2-1105 mutant (Figure 1C). In total protein samples, HvGBSSI protein was detected in the M2-1105 mutant, indicating that M2-1105 produced the HvGBSSI protein. To further localize HvGBSSI protein, the total proteins were divided into soluble proteins, which was present in the supernatant, and particle binding proteins that were in starch granules. HvGBSSI protein was only detected in the supernatant but not in starch granules, suggesting that the HvGBSSI protein in the M2-1105 mutant failed to bind to the starch granules.

### Two mutations present in the *Wx-1* gene in the M2-1105 mutant

**Figure 2:**
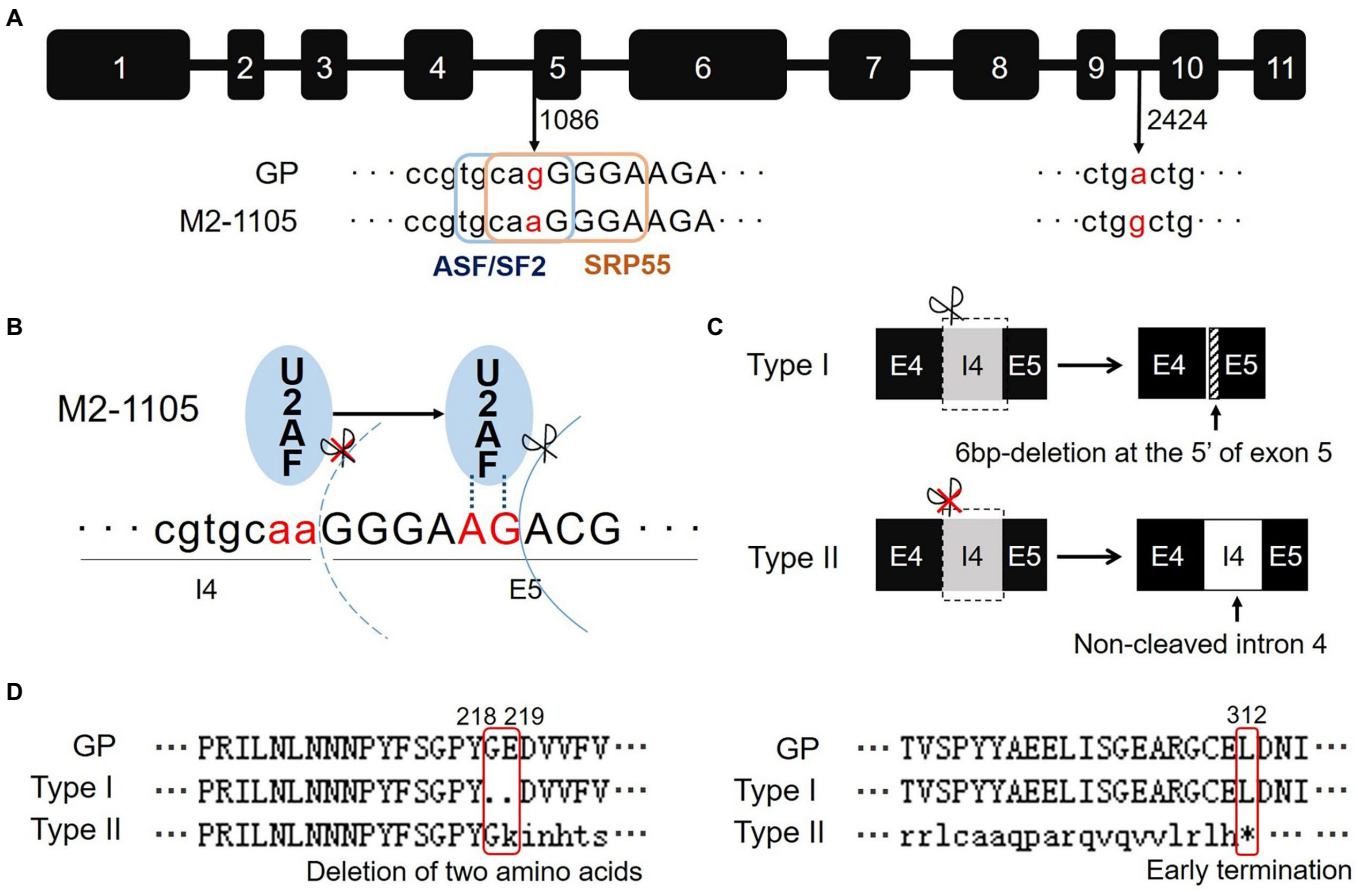
A single substitution G1086A in the *Wx-1* gene is responsible for the defective HvGBSSI phenotype in the M2-1105 mutant. **(A)** Sequence analysis of the *Wx-1* gene in Golden Promise and the M2-1105 mutant. Lowercase indicates intron and uppercase indicates exon. G1086A occurs at the cutting site of fourth intron and fifth exon. Two elements (ASF/SF2 and SRP55) were predicted using ESEfinder3.0. The mutation disrupted these two elements. **(B)** U2AF recognized the incorrect AG site in the exon 5. **(C)** Comparison of two types of *Wx-1* transcripts in the M2-1105 mutant. A 6-base deletion occurred in the 5′ end of exon 5 due to an incorrect recognition of an AG site right downstream of the mutant AA site. For the type II transcript, the fourth intron was not cleaved and an extra135 nucleic acids were present. **(D)** Protein sequence alignment of *Wx-1* cDNA sequences from parent line GP and the M2-1105 mutant. The type I had a deletion of G218 and E219. *indicates the termination caused by an early stop codon from the type II cDNA.

To further characterize the HvGBSSI mutation in the M2-1105 mutant, we amplified the genomic DNA fragment of *Wx-1* gene from GP and the mutant M2-1105 by PCR. The final sequences were determined to be 2,793 bp for both GP and the mutant M2-1105 line. However, the M2-1105 had two single-nucleotide mutations: one change was from G to A (G1086A) at the nucleic acid 1,086; the other one (A2424G) was from G to A at the nucleic acid 2,424 (Figure 2A; Supplementary Figure S2). G1086A was located at the end of the 3′ of fourth intron as part of the splicing receptor site. The ESEfinder3.0 program predicated that G1086A disrupted the exonic splicing enhancer (ESE) elements ASF/SF2 with sequence 5′-TGCAGG-3′ and SRP55 with sequence 5′-CAGGGGA-3′ in the mutant line. In contrast, A2424G was located in the middle of intron 9 and had no effect on the ESE elements.

### *Wx-1* transcripts in the M2-1105 mutant

We focused on the *Wx-1* transcripts in the M2-1105 mutant since the G1086A mutation occurred at the RNA splicing site. We sequenced 62 cDNA clones of *Wx-1* from the M2-1105 mutant and found that these cDNAs were from two types of transcripts. The type I transcript, which was represented by 55 clones, had a size similar to that of GP, while the type II, with 5 clones, was longer than the GP transcript (Figure 1D). Sequencing results showed that transcript lengths of type I and II were 1,806 bp and 1,948 bp, respectively, different from the length of 1,812 bp in GP. The type I transcript had a 6 bp deletion at the 5′ of fifth exon, resulting in a deletion of two amino acid glycine at 218 (G218) and glutamate at 219 (E219). In the type II transcript, the fourth intron was not cleaved, leading to a premature termination codon (TGA) at nucleic acids 994–996 and a truncated protein of 311 amino acids (Figures 2C,D; Supplementary Figures S2, S3).

We analyzed the consequences of the splicing-related mutation G1086A and the resulting mRNA variants. G1086A occurred at the 3′ splicing site of the fourth intron, changing the sequence from AG to AA. This substitution resulted in a failed binding to the 3′3′ splicing site by the U2AF factor. Subsequently, U2AF incorrectly bound to the AG sequence that was the 5th and 6th nucleic acids of the fifth exon, leading to a 6 bp deletion at the beginning of the fifth exon. This is consistent to the sequencing results of the Type I transcript (Figure 2B). On the other hand, as the mutation of G1086A led to the loss of the exonic splicing enhancer (ESE) elements (ASF/SF2 and SRP55), U2AF was not always able to recognize the AG site within the fifth exon, and thus, the type II mRNA with an intact fourth intron was produced. Based on this analysis and our results, we revealed that U2AF must not combine with the mutated 3′ splicing site of the fourth intron, but combine with the wrong AG site of fifth exon randomly, which resulting erroneous transcripts type I and II of *Wx-1* in M2-1105.

Further analysis of the expression levels of the two types of transcripts relative to *β-Actin* and *GAPDH* showed that the sum of the two transcripts was not significantly different from that of the wild type, but there was a huge difference between the two types of transcripts, with the expression level of the type I transcript was significantly higher than that of the type II transcript (Figure 1E), suggesting that U2AF prefers an AG site.

### Protein structure of the HvGBSSI variant in the M2-1105 mutant

**Figure 3:**
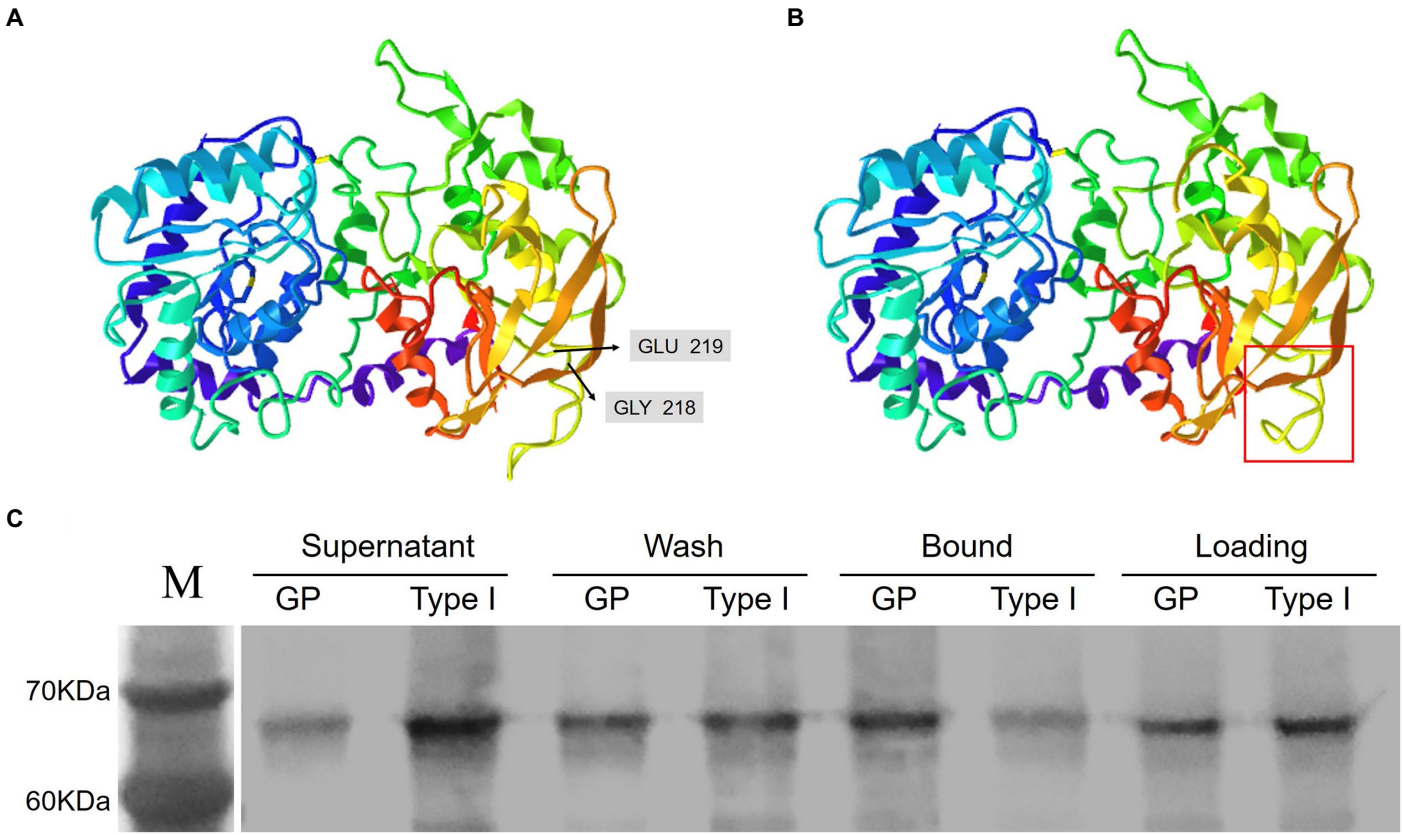
The HvGBSSI variant in the M2-1105 mutant was weakly associated with the starch granule fraction. **(A)** The predicted HvGBSSI structure of GP. **(B)** The predicted HvGBSSI structure of the typet I variant in the M2-1105 mutant. The boxed area highlights the affected β-sheet. **(C)** The raw starch-binding capacity of the recombinant HvGBSSI protein of GPGB and the type I variant of the M2-1105 mutant. Corn starch was incubated with purified recombinant HvGBSSI protein of GP and the type I variant of the M2-1105 line. The unbound HvGBSSI protein is designated the Supernatant fraction. The loosely bound GBSS1is eluted from the granules, after SDS washing and centrifugation, the supernatant is designated as the Wash fraction. The insoluble protein is designated as the Bound fraction. The Loading fraction indicates the equal amount of each recombinant protein per treatment.

Two different *Wx-1* transcripts in M2-1105 were translated to form two different protein types. The type II protein was terminated prematurely, likely with a complete loss of function. The type I transcript directs the expression of HvGBSSI mutant lacking two residues, G218 and E219. We hypothesized that the absence of two amino acids might affect the higher structure of the HvGBSSI protein in the M2-1105 mutant because only deletion of the two amino acids in the mutant should not change protein size too much. Both G218 and E219 were part of the β-pleated sheet inn the N-terminus and were not close to Adenosine 5′-Diphosphoglucose (ADPG) binding pocket of HvGBSSI. The deletion affected the polypeptide folding and subsequently formed a varied β-pleated sheet, leading to a conformational change leading to conformational change (Figures 3A,B).

### The HvGBSSI variant in the M2-1105 mutant has a decreased level of starch-binding capability

HvGBSSI is tightly bound to starch granules, and the binding to starch granules stimulates its enzymatic activity. We hypothesized that the type I protein in the M2-1105 mutant may have lost its ability to bind starch granules. To confirm, we conducted a starch binding assay. The *Wx-1* coding sequences of GP and the M2-1105 protein were expressed in the *E. coli Rosetta* strain (DE3; Supplementary Figure S4A). The recombinant proteins were purified to homogeneity (Supplementary Figure S4B) and incubated with 1% corn starch to determine their ability to bind to starch (Figure 3C). Our results showed that the amounts of the type I HvGBSSI protein were higher in the supernatant fraction but lower in the bound fraction than those of GP, supporting the notion that the binding capacity of the type I HvGBSSI variant was significantly reduced compared to GP.

### Comparison of starch granule morphology and size between the mutant line and parent line

**Figure 4:**
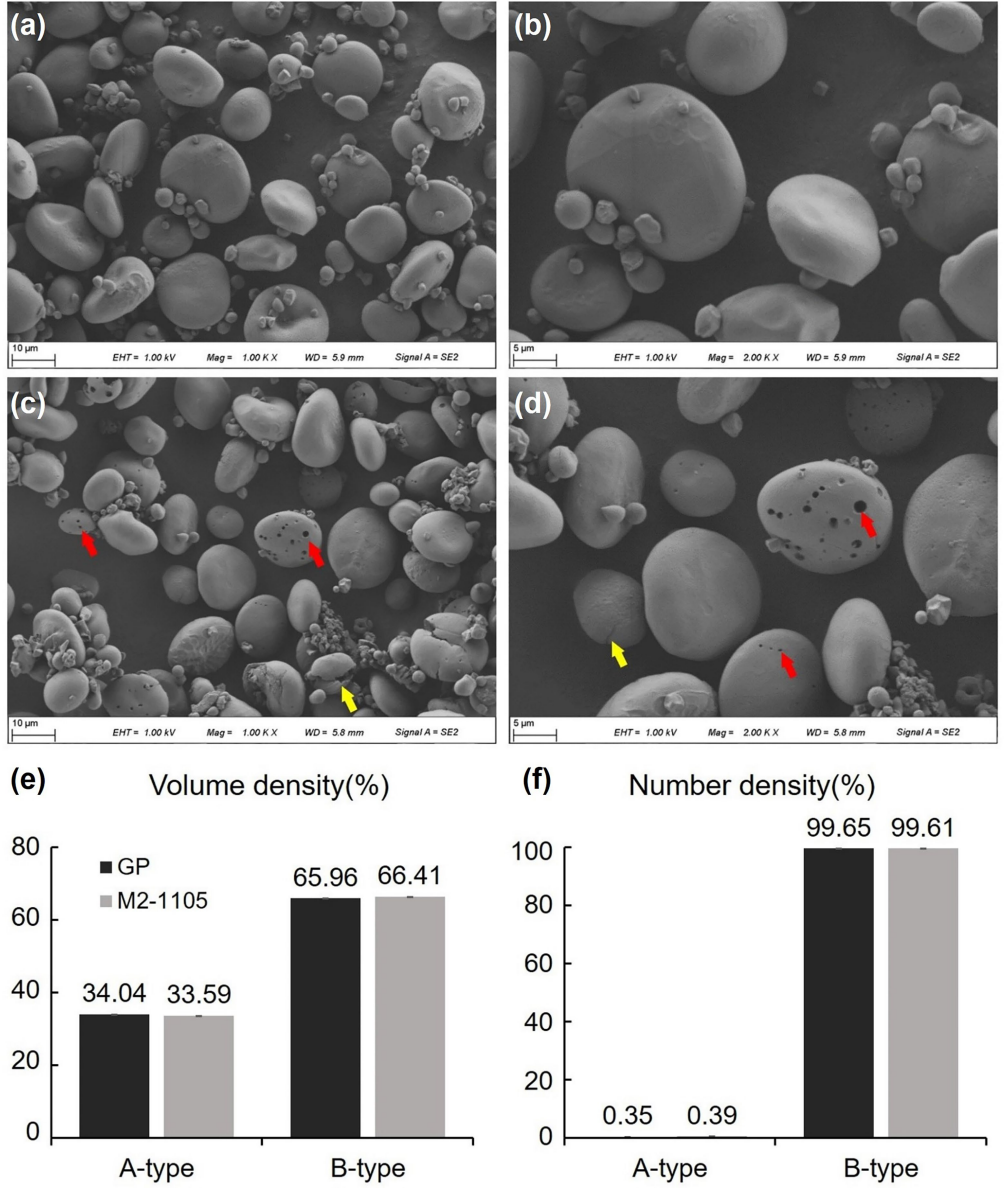
Characterization of starch granule morphology and particle size. Starch granule structures in the parental GP and the M2-1105 mutant line were analyzed *via* SEM. For each accession, at least three views were examined and similar results were observed. **(A)** Starch granule morphology of GP at 1,000× magnification. **(B)** Starch granule morphology of GP at 2,000× magnification. **(C)** Starch granule morphology of the M2-1105 mutant at 1,000× magnification. **(D)** Starch granule morphology of the M2-1105 mutant at 2,000× magnification. The red arrow indicates pores in the starch granules. The yellow arrow indicates broken starch granules. **(E)** The volume of A-type and B-type starch granules in GP and the M2-1105 mutant. **(F)** The number of A-type and B-type starch granules in GP and the M2-1105 mutant.

To evaluate the effects of the HvGBSSI sequence change on starch granule properties, we analyzed the starch granule morphologies of GP and the M2-1105 mutant using a scanning electron microscope (SEM). The SEM results showed that the M2-1105 starch granules had some pores on their surface, and some granules had broken edges compared to those of the parent line (Figures 4A–D). Further analysis of granule size by laser diffraction showed that start granules from GP and the M2-1105 mutant had no significant differences in the quantity or volume distribution of A-type and B-type starch granules (Figures 4E,F).

### Hvgbssi enzymatic activity, starch and protein contents of the M2-1105mutant

**Figure 5:**
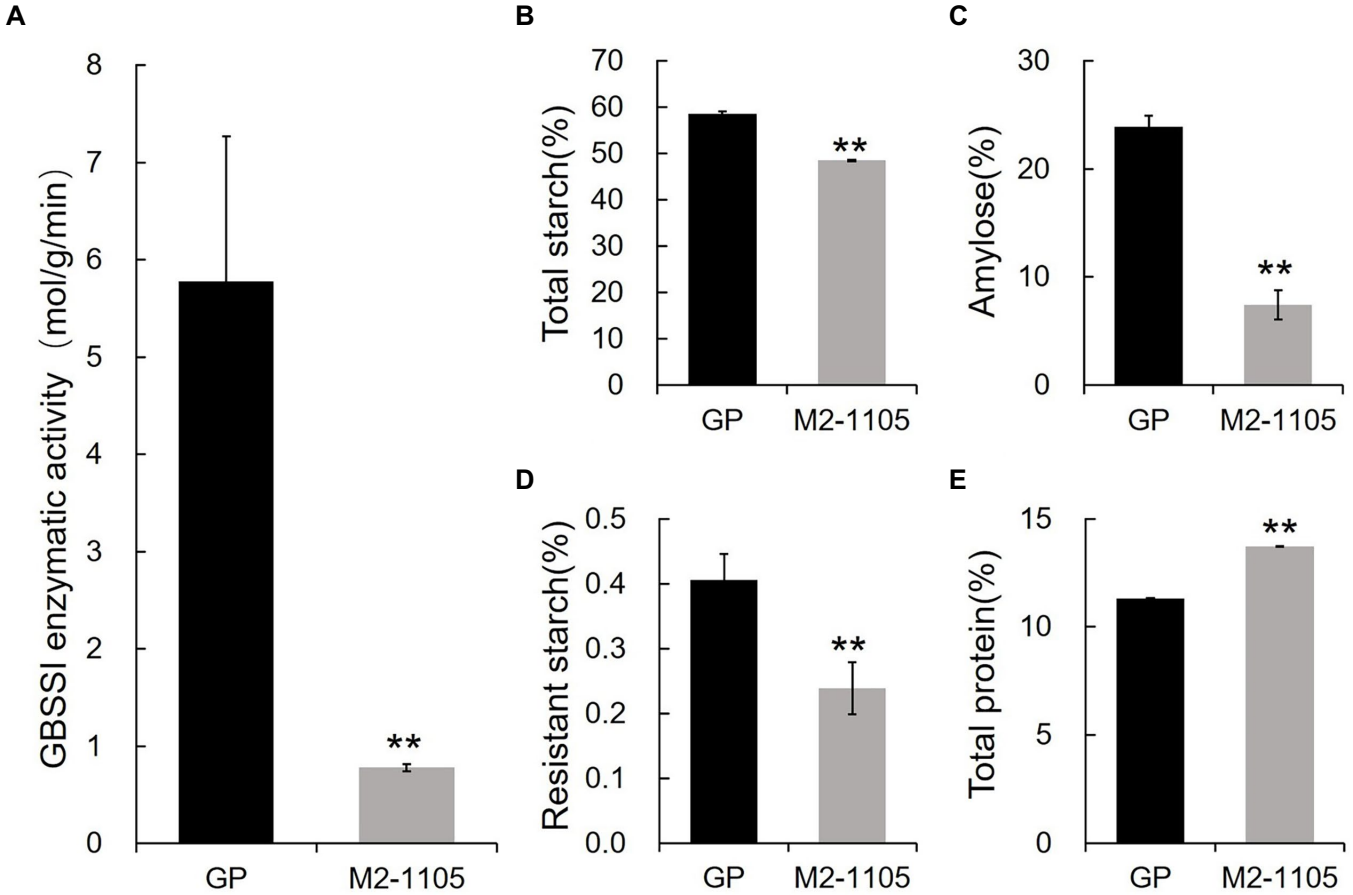
Enzymatic activity 15 days after flowering and starch characters in grains **(A)** The HvGBSSI enzymatic activity of GP and the M2-1105. **(B)** Total starch content; **(C)** Amylose content; **(D)** Resistant starch content **(E)** Total protein content of GP and the M2-1105. The data represent the mean of three replicates. **Highly significant difference (*p* < 0.01).

We further measured the enzymatic activity of wild-type and the type I variant of HvGBSSI using the purified recombinant proteins *in vitro*. The enzymatic activity of the type I HvGBSSI variant from the M2-1105 mutant was 0.778 mol/g/min, a dramatic decrease from that of GP at 5.78 mol/g/min (Figure 5A). Because HvGBSSI is the sole enzyme for amylose synthesis, we further analyzed the starch composition of grains in the M2-1105 mutant. There was a notable difference in the total starch, amylose, and resistant starch contents between GP and the M2-1105 seeds (Figures 5B–E). The contents of the total starch, amylose, and resistant starch in the M2-1105 mutant were 48.49%, 7.45%, and 0.24%, respectively, which were significantly lower than those of GP at 58.60%, 23.91%, and 0.41%, respectively. The protein levels in the mutant line were higher than those in GP, with M2-1105 at 13.71%, while GP was only 11.31%.

## Discussion

Ethyl methane sulfonate treatment is one of the most effective plant chemical mutagenesis techniques owing to its ability to increase the mutation frequency of genes compared with low natural mutation frequencies. Moreover, the favorable mutations can be identified rapidly, aiding in increasing the availability of different mutants. Presently, EMS mutagenesis is widely used in the construction of mutant banks and the creation of germplasm resources in several crops. In a previous study, EMS mutagenesis was used to obtain *SBEIIa* mutants, which had a significant increased amylose content (Botticella et al., 2011). In another study, *SSIIa* alleles created using EMS mutagenesis in soft wheat were evaluated for their effect on starch properties (Andrew et al., 2017). In the present study, we produced an EMS-mutagenized population of barley and identified a valid *Wx-1* mutation in M2-1105. The mutation led to a decrease in amylose content and is stably inherited in the offspring.

**Figure 6:**
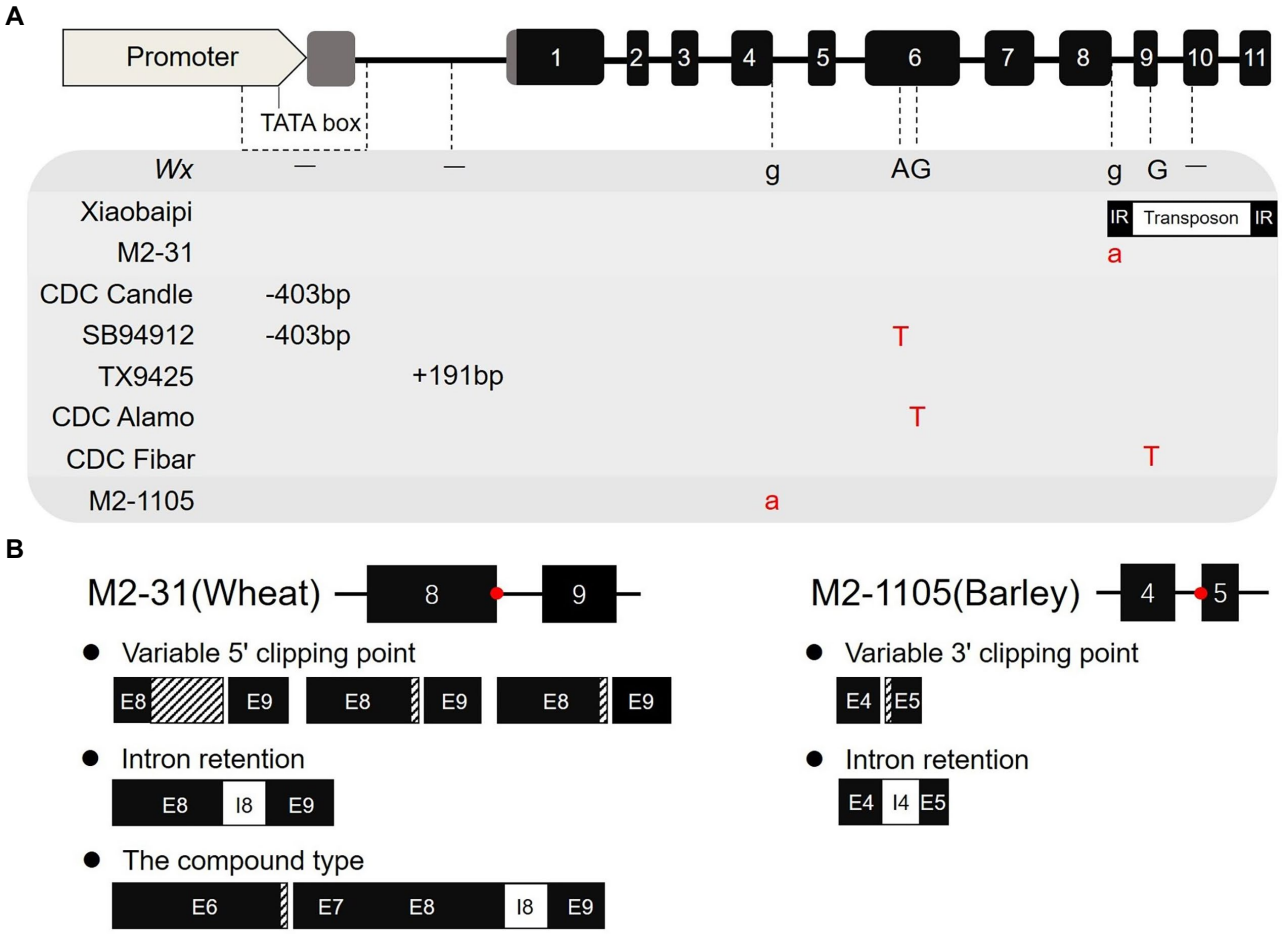
Variation types of the *Wx-1* gene in wheat and barley. **(A)** Comparison of *Wx-1* variation types in wheat and barley. There are two special variants of *Wx-1* in wheat and six other variants in barley. **(B)** Comparison of splice variation types between wheat *Wx-A1* mutant M2-31 and barley *Wx-1* mutant M2-1105.

In previous reports, some natural or artificial deletion mutations in *waxy* have been screened in wheat and barley (Figure 6A). For example, CDC Rattan, CDC Candle, and SB94912 have a 403 bp deletion in the promoter and 5′-UTR regions; TX9425 inserts 191 bases from the transcription start to start codon region; and the CDC Alamo and CDC Fibar sequences showed an SNP difference (Asare et al., 2012; Fan et al., 2017). All these mutations resulted in varying degrees of decreased amylose content. In our previous study, we identified a specific *Wx-1* gene variant in the Xiaobaipi accession that inserted a 2,178 bp transposon fragment in tenth exon, resulting in the deletion of the Wx-B1 protein (Zhang et al., 2017). The molecular mechanism of *Wx-1* inactivation in M2-1105, wherein a single-nucleotide change at the splicing receptor site leads to the production of abnormal transcripts, differs from previously reported mechanisms in barley and wheat.

The correct identification and removal of introns by the splicing machinery is a critical step in the gene expression process. Mutations that alter the sequence of splice sites or elicit splicing errors are often associated with loss of function. The alternative splice variants of one gene may encode a different protein, which in extreme cases can have an opposite function (Gehring and Roignant, 2020). The sequence change of a mononucleotide from G to A leads to the loss of an ESS site (TTTGGG) between the 3′ of exon 10 and the splice junction site of *SBEIIa*, resulting in two types of abnormal transcripts and an increase in amylose content (Bovina et al., 2015). In our recent study, we identified a *Wx-A1* deletion mutant (M2-31) in EMS mutagenic wheat population (Luo et al., 2019). *Wx-A1* in M2-31 mutated from G to A at the 5′ splicing site of intron 8 destroying the splicing donor site (GU). Moreover, it formed potential splicing regulatory motif ESEs with the capacity to inhibit splicing, thus resulting in five different types of abnormal transcripts (Figure 6B). In the present study, the splicing site of fourth intron was mutated from G to A in the mutant M2-1105 line. This mutation occurred at the 3′ splicing site, destroying the splice receptor site from GU to AG, which caused the U2AF factor to fail from binding to the AG site of fourth intron and discriminate to the incorrect AG site of fifth exon. In addition, this mutation also led to a decrease of cis-acting elements bound by the ASF/SF2 and SRP55 proteins. ASF/SF2 and SPR55 can help connect the 5′ and 3′ splicing sites and are also important SR that act through ESEs (pre-mRNA sequences; Wu and Maniatis, 1993; Black, 2003). Moreover, because of the absence of ASF/SF2 and SPR55, U2AF does not bind tightly to the incorrect AG site of fifth exon, which results in the production of two transcript types. However, this mutation did not affect the expression level of the *Wx-1* gene. Therefore, the destruction of both the 5′ splicing site and the 3′ splicing site produced incorrect transcripts, although it is possible that the destruction of the 5′ splice site had a greater impact.

Granule-bound starch synthase I belongs to the GT5 family of glycosyltransferases, and adopts a typical GT-B fold consisting of distinct N and C-domains (Coutinho et al., 2003). GBSSI activity is affected by its ADPG-binding and the starch-binding ability (Liu et al., 2009; Subasinghe et al., 2014). A previous study showed that a mutation at C487 of OsGBSSI, which was distant from the ADPG-binding, significantly decreased the starch adsorption ability of mutated OsGBSS1 compared with that of wild-type OsGBSSI (Liu et al., 2015). In our recent study, a single-base mutation in wheat *Wx-B1* resulted in an amino acid substitution (G470D) of GBSSI and disrupted the binding of *Wx-B1* to starch granules (Li et al., 2022). In the present study, the deletions of amino acid residues G218 and E219 of HvGBSSI in M2-1105, were not located in the ADPG binding pocket but rather in the β-sheets of the N-terminal domains (Leterrier et al., 2008). The binding interaction between protein and starch was mainly hydrophobic interaction, and also involved electrostatic interactions and hydrogen bonding (Xu et al., 2015). Charged residues were generally found in the binding sites in carbohydrate–protein and their direct interactions with carbohydrate made a major contribution to binding enthalpy (Fadda and Woods, 2010). Glycine and glutamate are both hydrophilic amino acids, but glutamate is a charged residue. We speculate that the deletion of glutamate at 219 of HvGBSSI might result in the decreased starch binding capacity of HvGBSSI. Through western blotting and the starch-binding ability assay, it was verified that the deletions of these two residues significantly decreased the starch binding ability of GBSSI in barley mutant line. This can explain the significant reduction of enzymatic activity of HvGBSSI in M2-1105. Another study also showed that the change of amino acid in CDC Alamo led to the waxy phenotype, which is associated with deficient targeting of HvGBSSI into starch granules (Hebelstrup et al., 2017). Further investigations are needed to identify other amino acid residues that influence the starch-binding capability of HvGBSSI.

In this study, the surface of part of the M2-1105 B-type starch granules had obvious pores, the similar holes were also observed on the starch granules of the waxy barley variety CDC Candle (Naguleswaran et al., 2013; Li et al., 2014). These pores on the granule surface might occur by sprouting in the field or during storage due to starch hydrolysis and increased enzymatic activity (Jensen and Heltved, 1982). Some studies have shown that the starch granules of barley, wheat, rice and maize are destroyed during germination, resulting in pore due to high α-amylase activity (Simsek et al., 2014; Zhang et al., 2020). Moreover, these pores on the granule surface also may be related to the enzymatic activation occurring while the barley grains were soaked during the extraction of starch granules (Higgins et al., 1976). Our previous studies indicated that there were no pores on the surface of starch granules of the hexaploid wheat line lacking *Wx-A1* when the grain samples were cut by knife, while there were some pores on the surface of starch granules of the hexaploid wheat line lacking *Wx-B1* when the grains were soaked in water to extract starch granules (Luo et al., 2019; Li et al., 2022).

In M2-1105, the loss of the HvGBSSI protein resulted in a decrease of total starch, amylose, and resistant starch contents and an increase in the total protein content. The increase in protein content was also found in previous studies. For instance, the protein content of the waxy line was reported to be higher than that of ordinary wheat (Caramanico et al., 2017). Waxy barley exhibits reduced water and nutrients loss during cooking, and decreased rate of starch coagulation and aging, indicating that the preservation of barley products can be greatly increased (Teixeira et al., 2017). The food processed with waxy barley has unique qualities, indicating its great potential for food development. The amylose content of M2-1105 was lowered to 7.45%. Thus, the M2-1105 mutant line of waxy barley may exhibit an altered end-use quality and provide a new germplasm resource for the creation of glutinous barley.

## Data availability statement

The data presented in the study are deposited in the DDBJ repository, accession number LC726273, LC726274, LC726275, LC726276, LC726277.

## Author contributions

QJ designed the experiments. GC, WH, QX, YaZ, HT, YJ, PQ, MD, JM, JW, GCh, XL, YW, and YoZ gave important suggestions to the research. XF, MR, QH, BW, HK, and JL performed experiments. XF analyzed the data and wrote the manuscript. All authors contributed to the article and approved the submitted version.

## Funding

This work was supported by the Sichuan Science and Technology Program, China, (2022ZDZX0014 and 2022YFH0055), and the International Science & Technology Cooperation project of Chengdu, Sichuan Province, China (2019-GH02-00078-HZ). Carlos Guzmán gratefully acknowledges the European Social Fund and the Spanish State Research Agency (Ministry of Science, Innovation, and Universities) for financial funding through the Ramon y Cajal Program (RYC-2017-21891).

## Conflict of interest

The authors declare that the research was conducted in the absence of any commercial or financial relationships that could be construed as a potential conflict of interest.

## Publisher’s note

All claims expressed in this article are solely those of the authors and do not necessarily represent those of their affiliated organizations, or those of the publisher, the editors and the reviewers. Any product that may be evaluated in this article, or claim that may be made by its manufacturer, is not guaranteed or endorsed by the publisher.

## Footnotes

^1^ http://krainer01.cshl.edu/tools/ESE3/

^2^ http://www.sbg.bio.ic.ac.uk/phyre2/html/page.cgi?id=index

## Supplementary material

SUPPLEMENTARY FIGURE S1 Image of mature barley seeds.

SUPPLEMENTARY FIGURE S2 Comparison of nucleotide sequences in *waxy* gene transcripts of mutant line and GP.

SUPPLEMENTARY FIGURE S3 Comparison of amino acid sequences of HvGBSSI protein of mutant line and GP.

SUPPLEMENTARY FIGURE S4 SDS-PAGE of HvGBSSI protein. **(A)** The prokaryotic expression of *Wx-1* of GP and M2-1105. The results indicated that M2-1105 type I encoded a protein with similar molecular weight to those of GP. **(B)** Purified recombinant protein of GP and the Type I protein of M2-1105.
