# Supplementary figures and images for: HvGBSSI mutation at the splicing receptor site affected RNA splicing and decreased amylose content in barley
Source: Front Plant Sci. 2022 Sep 23;13:1003333. doi: 10.3389/fpls.2022.1003333 (PMC9538149; doi:10.3389/fpls.2022.1003333)

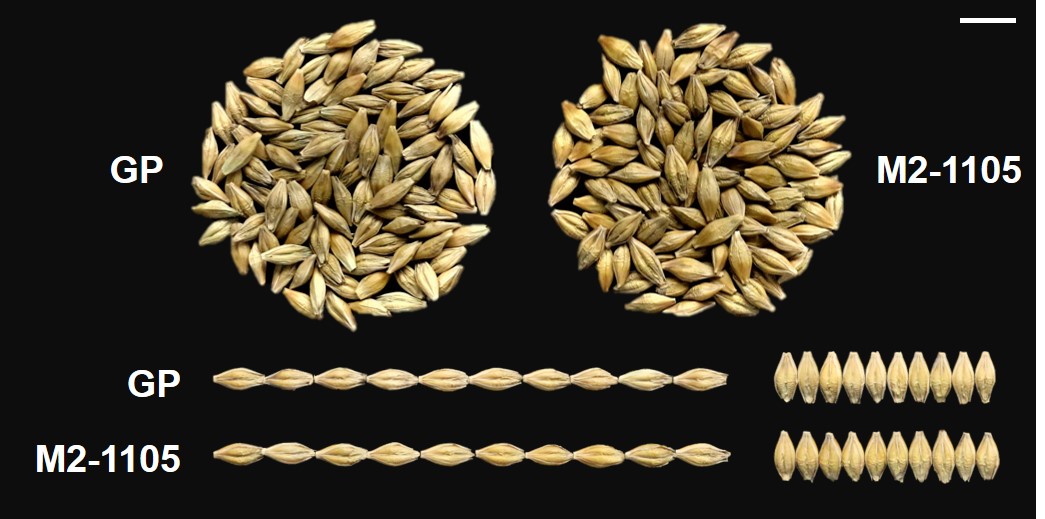

Supplement: SUPPLEMENTARY FIGURE S1 — Image of mature barley seeds. [file Image_1.JPEG]

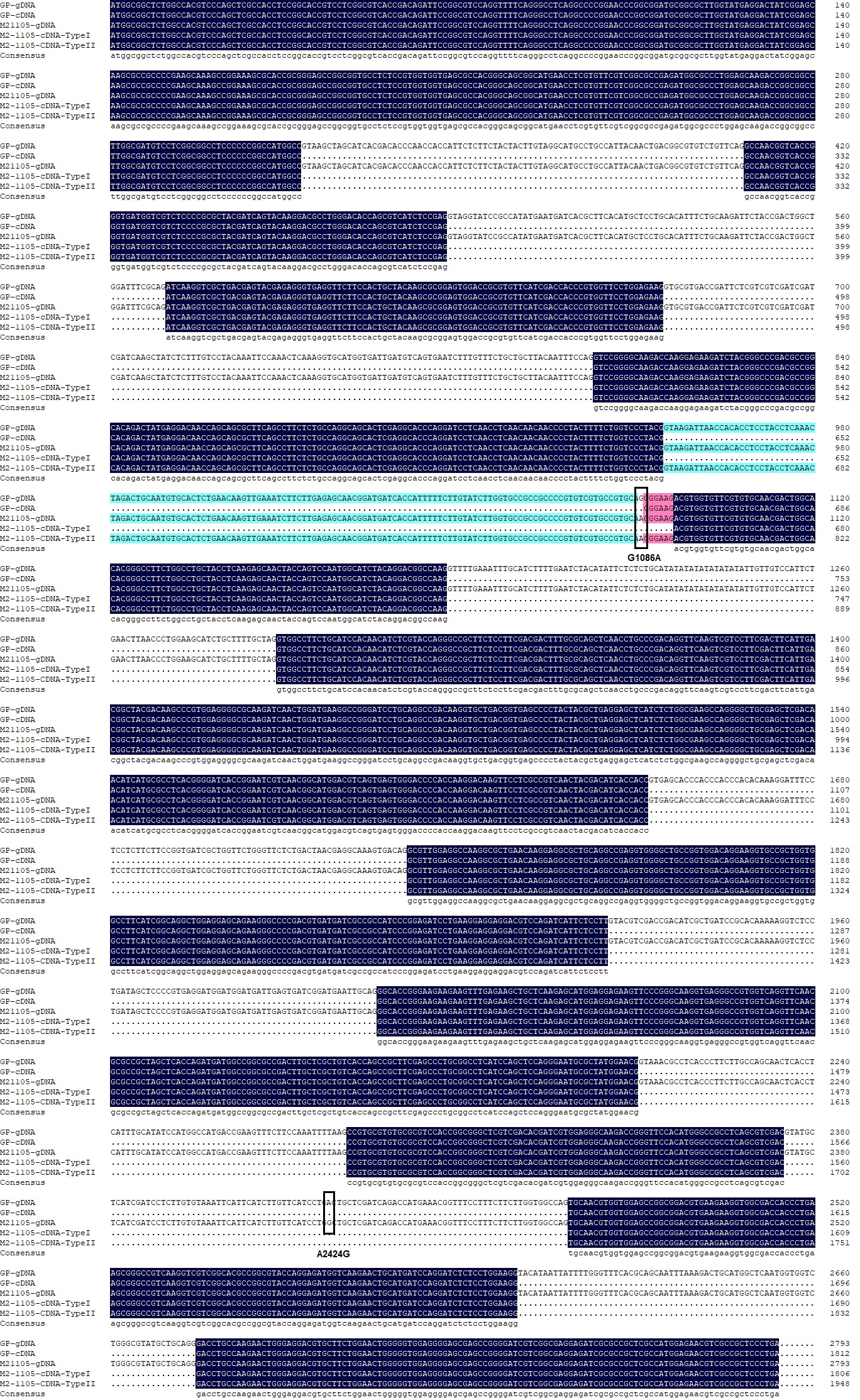

Supplement: SUPPLEMENTARY FIGURE S2 — Comparison of nucleotide sequences in waxy gene transcripts of mutant line and GP. [file Image_2.JPEG]

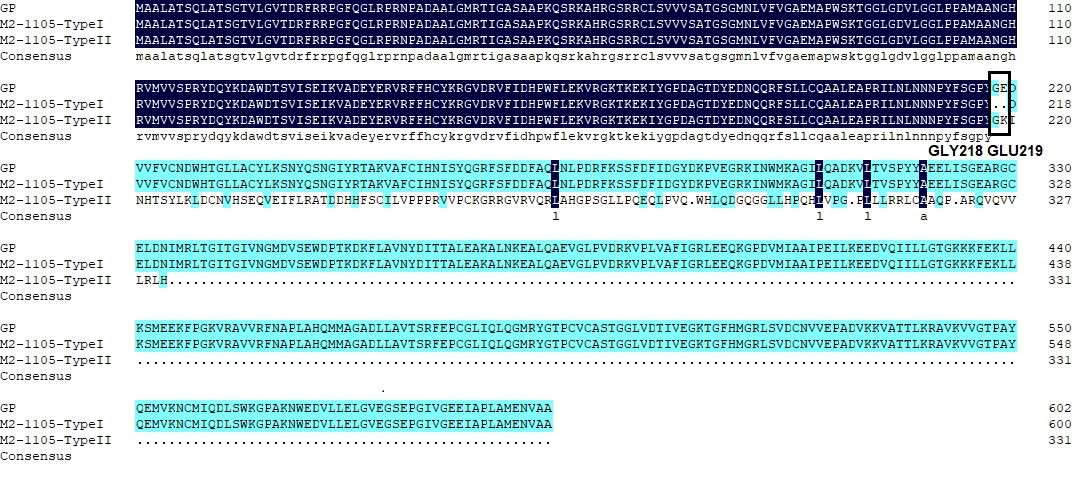

Supplement: SUPPLEMENTARY FIGURE S3 — Comparison of amino acid sequences of HvGBSSI protein of mutant line and GP. [file Image_3.JPEG]

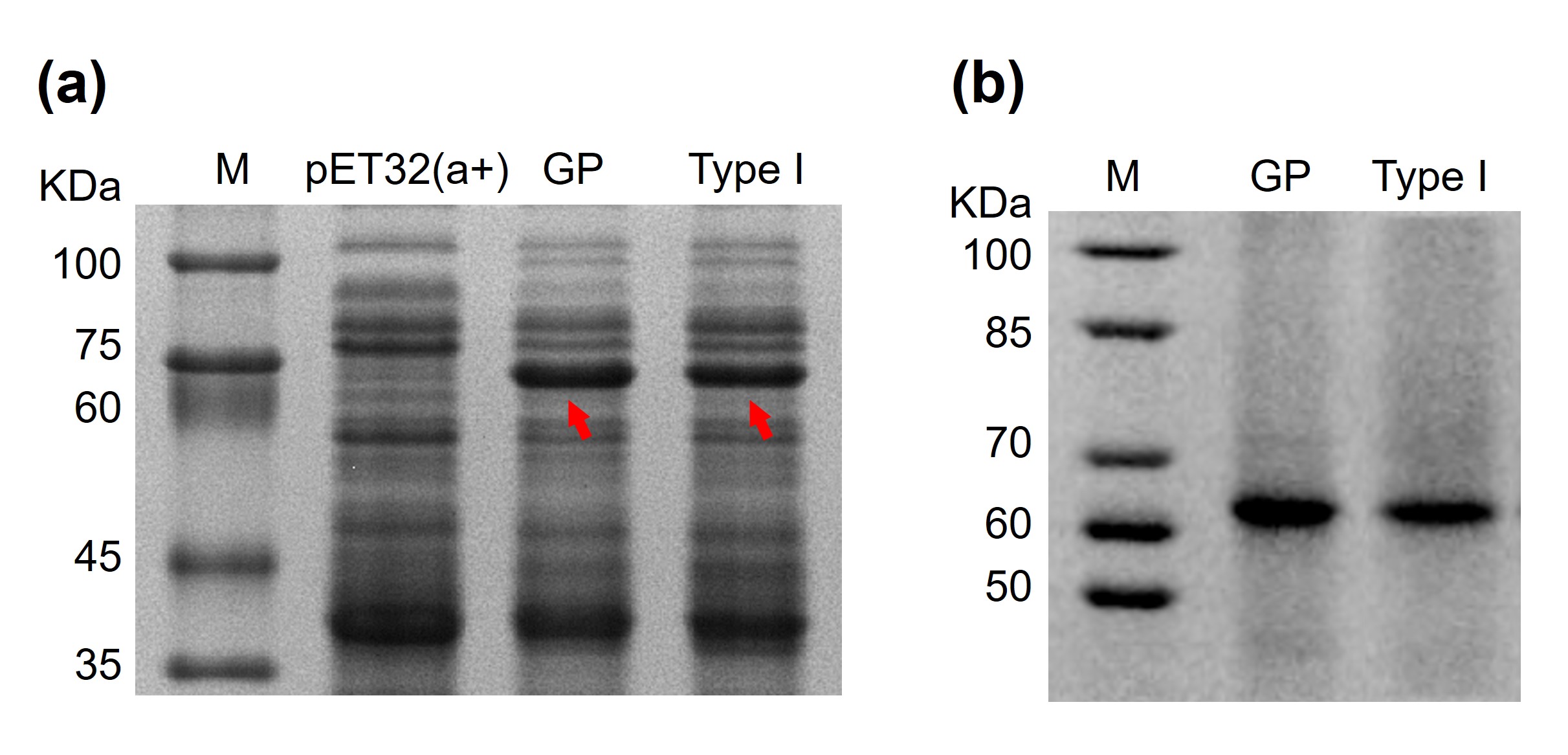

Supplement: SUPPLEMENTARY FIGURE S4 — SDS-PAGE of HvGBSSI protein. (A) The prokaryotic expression of Wx-1 of GP and M2-1105. The results indicated that M2-1105 type I encoded a protein with similar molecular weight to those of GP. (B) Purified recombinant protein of GP and the Type I protein of M2-1105. [file Image_4.JPEG]
