# Supplementary material for: HvGBSSI mutation at the splicing receptor site affected RNA splicing and decreased amylose content in barley
Source: Front Plant Sci. 2022 Sep 23;13:1003333. doi: 10.3389/fpls.2022.1003333 (PMC9538149; doi:10.3389/fpls.2022.1003333)
Supplement: Supplementary file 5 [file Table_1.DOCX]

**Table S1** Primer sequences

| Primer | Sequences |
| --- | --- |
| *Wx-1*-F | 5’-ATGGCGGCTCTGGCCACGT-3’ |
| *Wx-1*-R | 5’-TCAGGGAGCGGCGACGTTC-3’ |
| GP-*Wx-1*-qRT-F | 5’-ACCCAGGATCCTCAACCTC-3’ |
| GP-*Wx-1*-qRT-R | 5’-ACGAACACCACGTCTTCCC-3’ |
| M2-1105-TypeI-qRT-F | 5’-ACCCAGGATCCTCAACCTC-3’ |
| M2-1105-TypeI-qRT-R | 5’-CACCACGTCGTAGGGAC-3’ |
| M2-1105-TypeII-qRT-F | 5’-TAAGATTAACCACACCTC-3’ |
| M2-1105-TypeII-qRT-R | 5’-CTTGCACGGCACGACACG-3’ |
| Hv*Wx-1*-PE-F | 5’-TCGGATCCGAATTCGAGCTCATGGCGGCTCTGGCCACGTCC-3’ |
| Hv*Wx-1*-PE-R | 5’-TCGAGTGCGGCCGCAAGCTTTCAGGGAGCGGCGACGTTCTC-3’ |
